# Comparison of the Profile and Composition of Volatiles in Coniferous Needles According to Extraction Methods

**DOI:** 10.3390/molecules21030363

**Published:** 2016-03-17

**Authors:** Yonjin Jun, Sang Mi Lee, Hyun Kyoung Ju, Hong Jin Lee, Hyung-Kyoon Choi, Gyeong Suk Jo, Young-Suk Kim

**Affiliations:** 1 Department of Food Science and Engineering, Ewha Womans University, Seoul 120-750, Korea; ggumzo@hotmail.com (Y.J.); smlee78@ewha.ac.kr (S.M.L.); nia98@daum.net (H.K.J.); 2 Department of Food Science and Technology, Chung-Ang University, Anseong, Kyounggido 456-756, Korea; hongjin@cau.ac.kr; 3 College of Pharmacy, Chung-Ang University, Seoul 156-756, Korea; hykychoi@cau.ac.kr; 4 Horticultural Research Institute, Jeollanamdo Agricultural Research & Extension Service, Najusi, Jeollanamdo 520-715, Korea; hyeong21@korea.kr

**Keywords:** *Cupressaceae* family, coniferous needles, volatile composition, enantiomeric distribution, GC-MS, extraction methods

## Abstract

The enantiomeric distribution and profile of volatiles in plants, which affect the biological and organoleptic properties, can be varied depending on extraction methods as well as their cultivars. The secondary volatile components of the needles of three conifer cultivars (*Chamaecyparispisifera*, *Chamaecyparisobtusa*, and *Thujaorientalis*) were compared. Furthermore, the effects of three different extraction methods—solid-phase microextraction (SPME), steam distillation (SD), and solvent extraction (SE)—on the composition and enantiomeric distribution of those volatiles were elucidated. Monoterpene hydrocarbons predominated in all samples, and the compositions of sesquiterpenes and diterpenes differed according to the cultivar. In particular, the yields of oxygenated monoterpenes and sesquiterpenes were greatest for SD, whereas those of sesquiterpenes and diterpenes were highest for SE. On the other hand, more monoterpenes with higher volatility could be obtained with SPME and SD than when using SE. In addition, the enantiomeric composition of nine chiral compounds found in three cultivars differed according to their chemotype. There were also some differences in the yielded oxygenated monoterpenes and sesquiterpene hydrocarbons, but not monoterpene hydrocarbons, according to the extraction method. These results demonstrate that the extraction methods used as well as the cultivars influence the measured volatile profiles and enantiomeric distribution of coniferous needle extracts.

## 1. Introduction

*Chamaecyparispisifera* (CP), *Chamaecyparisobtusa* (CO), and *Thujaorientalis* (TO) conifers belonging to the *Cupressaceae* family are distributed mainly in Korea, Japan, and North America [1,2,3]. Members of the *Cupressaceae* family are all evergreen trees with similar needle-like leaves and a white stomatal line [4], and they are well known for their distinct aroma and for emitting phytoncides, which are antimicrobial volatile compounds [5]. The essential oil of the *Cupressaceae* species is used in a wide variety of products such as timber, infused tea, crude drugs, aromatherapy, deodorants, antioxidants, and antibacterial agents because of their specific aroma active compounds.

The secondary metabolites produced by the *Cupressaceae* family are mainly terpenes, such as α-pinene, β-pinene, and γ-terpinene [3,6], and these terpenes exhibit various biological activities, including antioxidant, anti-inflammatory, and anti-cancer properties. In general, plants with high monoterpene content are used for antioxidant and antibacterial agents, whereas those with high oxygenated terpene content are more effective as deodorants [1]. Moreover, the biological activities of those terpenes vary depending on their optical configuration, with their chirality influencing their threshold values [7,8,9]. The ratio of enantiomers can be used to evaluate the quality of foods and beverages with respect to their origin [10], manufacturing processes such as fermentation, drying, roasting, and alkali treatment, and aging during the shelf life [11]. For instance, (*R*)-(+)-limonene, which is a monoterpene hydrocarbon that is usually found in citrus peel oils [7,9], affects the nervous system, inducing increased systolic blood pressure, and subjective alertness and restlessness [6], and has stronger antibacterial and antifungal properties than (*S*)-(−)-limonene [6]. It is perceived as a fresh citrus and orange-like odor, with an odor threshold value of 200 ppb. Conversely, the (*S*)-(−)-isomer is described as having a harsh and turpentine-like note, with an odor threshold value of 500 ppb [12,13]. The enantiomeric distribution of volatile compounds has been developed with the recognition of enantio-differentiation as an important factor in those biological and organoleptic properties [14,15,16]. Accordingly, the investigation on the volatile profiles of the *Cupressaceae* family and the enantiomeric distribution of volatile compounds is crucially important to predict the biological activities depending on the *Cupressaceae* species.

Solid-phase microextraction (SPME) and solvent extraction (SE) are the predominant methods used in many studies to extract the volatile compounds [16,17,18,19]. SPME is particularly efficient for extracting highly volatile aroma compounds and can easily be used for sample preparation without requiring a solvent [20]. On the other hand, SE is dependent upon the solubility of the solvent and cannot avoid extracting non-volatile compounds such as fat, wax, and pigments, in addition to volatile compounds [21]. Steam distillation (SD) has been widely used for the analysis of essential oils [22,23,24], but some loss of water-soluble compounds does occur, and heat-labile compounds can be degraded during this process [22]. Accordingly, both the enantiomeric distribution and the profile of volatiles can be affected by the extraction method. However, there have been few investigations of how the enantiomeric distribution varies with the extraction method. Therefore, the aim of the present study was to determine the effects of three different extraction methods on volatiles and their enantiomeric distribution from the *Cupressaceae* family.

## 2. Results and Discussion

### 2.1. Differences of Volatile Compounds in Coniferous Needles According to the Extraction Methods

The variation in volatile compounds in coniferous needles with the cultivars was determined by analyzing the volatiles of three different coniferous needle samples using GC-MS and quantified using the internal standard method (Table 1, Table 2 and Table 3). In total, 114, 104, and 106 volatile compounds were found in CP, CO, and TO. The predominant compounds in all samples were terpenes, comprising monoterpenes (C10), oxygenated monoterpenes, sesquiterpenes (C15), oxygenated sesquiterpenes, and some diterpenes (C20). The profiles of terpenes were similar across cultivars, but their relative contents differed markedly according to cultivar (Table 2).

A total of 64, 60, and 68 volatile compounds were identified from CP, CO, and TO using SPME, respectively. Among the terpenes, monoterpenes were the most abundant volatile compound. The dominant compounds in the needles were (+)-α-pinene (mh4), β-myrcene (mh6), δ-3-carene (mh14), and (−)-bornyl acetate (mo23) in CP; β-myrcene (mh6), (+ or −)-sabinene (mh7), (+)-limonene (mh18), (−)-bornyl acetate (mo23), terpinenyl acetate (mo27), and stachene (dh3) in CO; and (+)-α-pinene (mh4), δ-3-carene (mh14), (−)-β-phellandrene (mh19), and (−)-caryophyllene (sh13) in TO.

The essential oils of CP, CO, and TO comprised 64, 62, and 75 volatile compounds, respectively. The SD method indicated that the most abundant volatiles in CP and TO were monoterpenes, whereas there was a greater proportion of oxygenated monoterpenes than monoterpenes in CO. The major monoterpenes were mh4, mh6, mh14, and mo23 in CP; mh7, mh18, mo23, mo27, dh3, and elemol (so2) in CO; and mh4, mh14, mh19, mo27, α-humulene (sh20), and spathulenol (so5) in TO.

Application of the SE method yielded 68, 75, and 70 volatile compounds in CP, CO, and TO, respectively. The most abundant volatiles were monoterpenes in both CP and CO, while the most abundant were diterpenes in TO. The predominantly detected volatiles were mh4, mh6, mo23, dh3, eudesma-4(14),11-diene (sh40), and α-phellandrene (mh13) in CP; mo27, so2, dh3, and rimuen (dh1) in CO; and mh4, mh13, mh19, sh13, sh20, and so5 in TO. The major volatile compounds in each cultivar, regardless of the method of extraction, were mh4 and mh6 in CP, mo27 and dh3 in CO, and mh4 and mh19 in TO.

The other volatile components, such as acids, alcohols, benzenes, ketones, aldehydes, esters, and hydrocarbons, were determined to be minor compounds in all samples. Among these, C6 alcohols and carbonyls such as 3-hexen-1-ol (al3), 2-cyclohexen-1-ol (al4), 2-cyclohexen-2-ol (al5), hexanal (c2), 2-methyl-2-pentenal (c3), 2-hexenal (c4), and 2,4-hexadienal (c5) were detected in the samples.

The major constituents obtained were similar for SPME and SD, while those obtained with SE differed significantly from the other two methods. The predominant volatiles in all samples were monoterpene hydrocarbons. In addition, the compositions of sesquiterpenes varied markedly among the three needle samples, although the contents were relatively low compared with monoterpenes in all samples. In this study, the major sesquiterpenes were sh40 in CP; so2 in CO; sh13 in TO. Also, the relatively high contents of β-cedrene (sh9) and sh20 were determined in TO. On the other hand, (+)-β-caryophyllene (sh14) was detected and positively identified using an authentic standard compound in this study. According to the previous studies, this unusual (+)-β-caryophyllene occurred in *Pellia endiviifolia*, *P*. *epiphylla* and *Metzgeria conjugate* [12,17]. In CO and TO, (+)-β-caryophyllene was found with its isomer, α-humulene (sh20) [26]. Among oxygenated sesquiterpenes, elemol (so2) having green odor was largely detected [21,22].

After determining the volatile compounds based on cultivars, the three different extraction methods (SPME, SD, and SE) were compared to elucidate the influence of extraction method on the volatile profiles (Table 1, Table 2 and Table 3). Prior to GC-MS analysis, it is usually necessary to prepare the samples using methods such as extraction and concentration in order to obtain more purified extracts. In addition, it is important to eliminate any interfering matrix to improve the detection limits for the specific compounds [25]. This has prompted the development of several extraction methods for sample preparation [16,20]. The volatiles extracted by the three extraction methods differed significantly not only with respect to their profiles, but also in their contents (Table 1, Table 2 and Table 3).

With regard to the extraction conditions, the profiles and contents of volatiles extracted using SD differed significantly compared to the other two methods, possibly due to the use of high temperatures. More oxygenated monoterpenes were included in the essential oils extracted using SD (CP, 12.68%; CO, 31.33%; TO, 7.29%) than in those extracted using SPME (CP, 10.88%; CO, 13.66%; TO, 3.81%) or SE (CP, 10.66%; CO, 8.44%; TO, 5.25%). Also, oxygenated sesquiterpenes having high molecular weights were detected in the essential oils extracted using SD or SE. However, those were absent in extracts by the SPME method. Previous studies have shown that long-term exposure to high temperatures can cause oxygenation of unsaturated sesquiterpene hydrocarbons during SD [27], although the use of higher temperatures (of 70 °C and 90 °C) provides better extraction of oxygenated sesquiterpenes (relative to lower temperatures of 25 °C and 35–50 °C) by enhancing the volatility of compounds [28].

There was less sabinene hydrate (mo4), which is unstable, and more mo15 in distillates of CP and CO than in that of TO. This could be explained by (–)-4-terpineol being thermally transformed from sabinene hydrate during SD at high temperatures [19]. Linalool (mo5) could be formed by the thermal degradation of linalyl acetate (mo12) during SD [1]; however, significant thermal transformation from mo12 to mo5 was not observed in the present study.

On the other hand, the profiles of volatiles obtained in SPME could be affected by both molecular weight and polarity—which are highly related to compound volatility—affecting the adsorption of volatiles on fibers in the headspace [27]. SPME can be a useful tool for analyzing volatile compounds in various food matrices and plants relatively easily without using any solvent and be considered as complementary to other extraction methods such as SE and SD, focusing on highly volatile compounds [27,28,29]. In the present study, oxygenated sesquiterpenes with relatively high molecular weights (with the exception of so4 and so16) were detected in the extracts yielded by both SD and SE, but absent in that yielded by SPME (Table 3). Furthermore, highly volatile compounds such as monoterpene hydrocarbons were yielded mainly by SPME and SD, whereas more sesquiterpenes and diterpenes were found following SE. These results could be explained based on the findings of the Richter and Schellenberg study [19], which demonstrate that the volatility of compounds is a critical factor for SPME and SD, and that the SE method can be influenced by the solubility of volatiles in their solvent system.

### 2.2. Enantiomeric Distribution of Terpene Isomers

Enantiomeric configuration and ratio are important in terms of organoleptic and biological properties [8,29]. Also, the enetiomeric distribution could be effectively applied to the recognition of honey authenticity [30]. In the present study, the optical isomers were separated into individual components at different chromatographic retention times using an enantioselective column. In particular, the following nine compounds were determined as enantiomeric isomers using a Cyclodex-B column: α-pinene, sabinene, limonene, β-phellandrene, 4-terpineol, bornyl acetate, γ-muurolene, caryophyllene, and γ-curcumene. Chirality was identified for all of these enantiomers except for sabinene, γ-muurolene, and γ-curcumene, using authentic chiral standards or retention indices. All (*S*)-(−)-isomers identified using Kovats retention index and/or positive identification were eluted prior to their corresponding (R)-(+)-isomers.

The enantiomeric distribution of the nine chiral compounds in the three cultivars differed according to their chemotypes and according to the extraction method used with respect to the contents of oxygenated monoterpenes and sesquiterpene hydrocarbons, but not monoterpene hydrocarbons (Table 4).The enantiomers of monoterpene hydrocarbons were (−)-α-pinene (mh3), (+)-α-pinene (mh4), (+ or −)-sabinene (mh7), (+ or −)-sabinene (mh10), (−)-limonene (mh17), (+)-limonene (mh18), (−)-β-phellandrene (mh19), and (+)-β-phellandrene (mh20). In all cultivar samples, the predominant isomer of α-pinene was expressed as the (+)-configuration (mh4). Its enantiomeric excess was high in all samples, with the highest optical purity being observed for CO. Both mh7 and mh10 were only detected in CO and TO. Unfortunately, enantiomers of sabinene could not be positively identified based on the authentic chiral standard compounds used, and hence the predominant chirality could not be determined. The enantiomeric excess value of sabinene was 100%in CO, while it was below 50% in TO regardless of the extraction method. Furthermore, the optical configuration of limonene was not affected by the extraction method in all samples investigated, and a higher enantiomeric excess value was observed in CO for (+)-isomer (91.52%–97.48%). The chiral activities of limonene, which is one of the major compounds in *Anethum sowa*, have been studied for psychological effects such as antibacterial and antifungal properties. (*R*)-(+)-Limonene induced an increase in systolic blood pressure with subjective alertness and restlessness of the nervous system, whereas inhalation of (−)-isomer led to increased systolic blood pressure with no effects on psychological activity [8]. In terms of their odor properties on configuration, (+)-limonene is associated with the orange citrus odor of the *Citrus* species, while its (−)-isomer gives off the minty odor note of the *Mentha* species [25]. Zawirska-Wojtasiak and Wasowicz found that the enantiomeric ratios of chiral monoterpene hydrocarbons such as α-pinene, camphene, β-pinene, and limonene did not differ significantly between using SD and SPME [29]. Consistent with that study, the enantiomeric excess values of α-pinene and limonene in all cultivar samples in this study were not affected by the extraction method used. On the other hand, in the present study, the enantiomer distribution of β-phellandrene in CP differed between SD and SE. Interestingly, β-phellandrene was present as the (+)-form with low optical purity in the essential oils from CO, whereas the predominant isomer was the (–)-form in the other samples studied. Consequently, the differences of enantiomeric excess for monoterpene hydrocarbons, except for β-phellandrene, in three coniferous needle samples were not influenced by extraction methods.

Unlike monoterpene hydrocarbons, the enantiomeric excess of oxygenated monoterpenes (e.g., (−)-4-terpineol (mo15), (+)-4-terpineol (mo16), (−)-bornyl acetate (mo23), and (+)-bornyl acetate (mo24)) in samples differed among the three extraction methods. In CP and TO, the optical purity of 4-terpineol was diminished when SD and SE were applied. Moreover, the enantiomeric excess of bornyl acetate in all samples was slightly reduced in extracts with SD or SE. Regarding the extraction conditions, differences in the enantiomeric excess of oxygenated monoterpenes have also been found in previous studies of the enantiomeric distribution [11,30]. According to a previous study, partial or total racemization could occur depending upon the temperature and pH value of the medium, and, in particular, the neutral medium (pH 6.9 and pH 6.3) keeps their strong enantiomeric excess unchanged [30]. Also, the enantiomeric distribution of distilled lime oils showed similar results in 4-terpineol and α-terpineol as well as linalool, possibly due to acid-catalyzed reaction [31]. Thus, acidic or thermal conditions and rearrangements during SD or SE can cause the racemization of oxygenated monoterpenes. In the present study, the relatively reduced enantiomeric excess of 4-terpineol and bornyl acetate with SD and SE may have been due to the occurrence of thermally induced rearrangements.

A higher enantiomeric excess was observed for sesquiterpenes in SD: (+/−)-γ-muurolene (sh10 and sh12 in CO), (+/−)-caryophyllene (sh13 and sh14 in both CP and CO), and (+/−)-γ-curcumene (sh16 and sh19 in both CP and TO). The enantiomeric excess of sh10 and sh12 in all samples was decreased with SE relative to SD and SPME, while that in the essential oil of CO exhibited strong purity, with an enantiomeric excess value of 100. The enantiomeric excess values of sh13 and sh14 in CP and CO were higher for SD than for either of the other two extraction methods. Interestingly, the predominant enantiomer of caryophyllene was expressed as the (+)-form only in the extracts of CO, unlike that in extracts from the other samples. Consequently, the enantiomeric excesses of sesquiterpenes in all cultivar samples were influenced by extraction methods. That is, the change of enantiomeric ratio and configuration by extraction method could be used to evaluate biological and organoleptic properties. (+ or −)-γ-Curcumene (Sh16) and (+ or −)-γ-curcumene (sh19) were only detected in both CP and TO when using SPME and SD. The enantiomeric excess values of γ-curcumene with SPME were 52.10% and 51.40% in CP and TO, respectively, while that with SD was 100% for both CP and TO. The occurrence of both enantiomers of sesquiterpenes has not been widely reported [17], because it is labile and prone to rearrangement [32]. Isomeric sesquiterpenes are formed from germacrene enantiomers through thermal, photochemical, or acid-catalyzed rearrangements [32]. Furthermore, unstable sesquiterpenes are expected to form from single enantiomers by isomerization or rearrangement during SD [32]. Therefore, the increased enantiomeric excess of γ-muurolene, caryophyllene, and γ-curcumene found in the present study could be due to the lability of sesquiterpenes.

## 3. Experimental Section 

### 3.1. Plant Materials

Needles of conifer (*Chamaecyparispisifera*, *Chamaecyparisobtusa* and *Thujaorientalis*), which were cultivated in Jeollanam-do and Chungcheongbuk-do, Korea in 2012 and 2013, were harvested at the fruiting stage. All needle samples were transported after cutting several branches off the tree. Then, the green branchlets were chopped from the woody twigs. They were fresh frozen and stored at −70 °C in a deep freezer prior to the extraction of volatile compounds.

### 3.2. Chemicals

The volatile compounds which were used for positive identification were purchased from Sigma-Aldrich (St. Louis, MO, USA), except for α-terpineol, which was supplied by Wako Pure Chemical Industries (Osaka, Japan) and Samchun Chemical (Ansan, Gyeonggi-do, Korea), respectively. Dichloromethane was obtained from JT Baker Chemical Co. (Phillipsburg, NJ, USA).

### 3.3. Extraction of Volatile Compounds

Three different extraction methods—solid-phase microextraction, steam distillation and solvent extraction—were conducted in order to obtain volatile profiles of coniferous needles.

#### 3.3.1. Solid-Phase Microextraction (SPME)

Coniferous needles were rapidly frozen using liquid nitrogen to inactive enzymes and about 1 cm cut off. Each of the cut needle samples (5.0 g) was put into a 60 mL vial and 100 μL of 2-octen-1-ol (6.00 × 10^2^ μg/mL in dichloromethane) was spiked as an internal standard (ISTD), respectively. After that, the vials were sealed using aluminum caps with PTFE/Red Rubber septa (Supelco, Bellefonte, PA, USA) and kept at 40 °C for 30 min to reach an equilibrium state. Volatiles were adsorbed on SPME fiber which was coated with 50/30 μm divinylbenzene/carboxen/polymethylsiloxane (DVB/Carboxen/PDMS) (Supleco, Bellefonte, PA, USA) for 15 min and desorbed at 250 °C for 5 min in a GC injection port. For desorption, cryo-focusing, dipping the beginning part of column into liquid nitrogen, was performed. All experiments were conducted in triplicate.

#### 3.3.2. Steam Distillation (SD)

Soaked into 1 L distilled water at 4 °C for 48 h were 200 grams of needles; the extraction was followed by steam distillation using Clevenger-type apparatus [1,28]. Before steam distillation, 2 mL of ISTD solution (1.00 × 10^5^ μg/mL in dichloromethane) and extra 1 L of distilled water were added into the sample solutions. After that, distillation was conducted for 5 h and, subsequently, hydro-distilled essential oil separated from the aqueous layer by centrifugation twice at 3000 rpm, at 4 °C for 20 min. The obtained essential oils were diluted ten to one with dichloromethane for GC-MS analysis. Steam distillation of samples was conducted in triplicate.

#### 3.3.3. Solvent Extraction (SE)

Volatiles in needles of conifer (20.0 g) were extracted with 100 mL dichloromethane. After adding the ISTD solution (1 mL, 6.00 × 10^2^ μg/mL in dichloromethane), the extracts were magnetically stirred at 300 rpm for 90 min. The dichloromethane layer was separated from the aqueous layer and washed with distilled water twice. Then, the solutions were dehydrated with anhydrous sodium sulfate (Sigma-Aldrich) on 110 mm filter paper (Hyundai Micro Co., Seoul, Korea). The oleoresin was concentrated to a final volume of 0.3 mL under a gentle stream of nitrogen gas. All extraction was conducted in triplicate.

### 3.4. Gas Chromatography-Mass Spectrometry (GC­MS) Analysis

HP 6890N gas chromatograph coupled with 5975A mass selective detector (Agilent Technologies, Palo Alto, CA, USA) was used to analyze the volatiles in coniferous needles. Extracts obtained from three cultivars were separated on a Cyclodex-B column (30 m length × 0.25 mm i.d. × 0.25 μm film thickness, J & W Scientific, Folsom, CA, USA). As a carrier gas, helium constantly flowed at a rate of 0.8 mL/min. GC oven temperature was initially kept at 40 °C for 5 min, and elevated to 75 °C at a rate of 10 °C/min, and increased to 90 °C at 2 °C/min, and raised to 120 °C at 5 °C/min, and reached to 200 °C at 3 °C/min, and then held for 15 min at the final temperature. Injector was kept at 250 °C and 1 μL of the samples was injected in the split mode. The split ratio was set at 20:1 for SPME and 50:1 both for SD and SE, respectively. The temperatures of auxiliary channel and ion source were 250 °C and 230 °C, respectively. Volatiles transported into MS were scanned at a range from 35 to 550 a.m.u with a scan rate of 2.83 scan/s in electron ionization (EI) mode (70 eV).

### 3.5. Identification and Quantification of Volatile Compounds

Volatile compounds in coniferous needles with the cultivars was determined by analyzing the volatiles of three different coniferous needle samples (*Chamaecyparispisifera*, CP; *Chamaecyparisobtusa*, CO; *Thujaorientalis*, TO). Each volatile compound was positively identified by comparing both its mass spectral data and retention index with those of an authentic standard. When authentic standards were not available, each volatile compound was tentatively identified by comparing with computer library (Wiley 7n.L) (Hewlett-Packard Co., Palo Alto, CA, USA, 1995) and Kovats retention index (RI) value in the literature [16,33,34]. The RI values were calculated with n-alkane (C7-C22) as external standards. Volatile compounds were quantified with an internal standard method which was calculated with the relative peak ratio of their peak areas to that of internal standard. All experiments were performed in triplicate and results were presented as average ± standard deviation of in dependent triplicate data.

### 3.6. Assessment of Enantiomeric Excess

Enantiomeric excess, a measure of optical purity, was calculated as follows [12,16];

(1) $$\text{enantiomer excess }(\text{e.e},\%)=\frac{\left(\text{predominant enantiomer}-\text{minor enantiomer}\right)}{\left(\text{predominant enantiomer}+\text{minor enantiomer}\right)}\times 100$$

The excess of predominant enantiomer was present with its configuration. The enantiomeric excess value of 100 indicates high purity of the single enantiomer, contrary to that of 0 for racemates.

### 3.7. Statistical Analyses

Analysis of variance (ANOVA) was performed with general linear model procedure in SPSS (version 12.0, Chicago, IL, USA) to evaluate significant differences of volatile compounds in samples. *Post-hoc* analysis was determined using Duncan’s multiple comparison test (*p* < 0.05).

## 4. Conclusions

The contents and composition of volatiles in the three cultivars were investigated by three different extraction methods and they were significantly affected by both the cultivars and the extraction method used. In particular, oxygenated monoterpenes and oxygenated sesquiterpenes were determined in high abundance following SD, possibly due to the oxygenation of unsaturated terpenes. Most diterpenes in all samples were detected following extraction by SD or SE, respectively, with stachene (dh3) being the major diterpene hydrocarbon. Monoterpene hydrocarbons with high volatility were more readily detected by SPME than by SE. These observations indicate that the extraction method used influenced the measured volatile profiles of our samples. Furthermore, in the present study, the various effects of the three extraction methods on enantiomeric distribution have been explained in terms of enantiomeric excess. The enantiomeric purity of oxygenated monoterpenes tended to be relatively reduced when using SD and SE, whereas that of sesquiterpene hydrocarbons tended to be relatively increased when using SD; these differences in purity of the constituents of pine needle extracts could affect the biological and organoleptic properties of the volatile compounds obtained from there. Therefore, the changes in enantiomeric distribution according to extraction methods should be considered when applied to the evaluation of organoleptic and biological activities as well as the authenticity and adulteration of plants and their products. The extraction method with its minimal change would be most appropriate in terms of natural identical properties.

## Figures and Tables

**Table 1:** Volatile aliphatic and aromatic hydrocarbons of three different cultivars based on different extraction methods.

Relative Peak Area (Mean ± SD) ^3^							
No.	RI ^1^	Volatile Compounds ^2^	Extraction Methods	Cultivar Species			ID ^4^
				CP	CO	TO	
**Acids**							
a1	857	Acetic acid (64-19-7) ^7^	SPME SD SE	0.009 ± 0.001 a ^5^ nd ^6^ a nd a	0.023 ± 0.004 b nd a nd a	0.107 ± 0.008 c nd a nd a	C
a2	1140	Butanoic acid (107-92-6)	SPME SD SE	nd a nd a nd a	nd a nd a 0.062 ± 0.012 b	nd a nd a nd a	C
**Alcohols**							
al1	852	1-Penten-3-ol (616-25-1)	SPME SD SE	0.034 ± 0.004 b nd a 0.052 ± 0.006 b	0.042 ± 0.006 b nd a nd a	nd a nd a nd a	C
al2	964	2-Penten-1-ol (20273-24-9)	SPME SD SE	nd a nd a 0.030 ± 0.007 c	nd a nd a 0.017 ± 0.002 b	nd a nd a nd a	C
al3	1041	3-Hexen-1-ol (928-96-1)	SPME SD SE	0.017 ± 0.007 a nd a 0.021 ± 0.018 a	0.118 ± 0.019 b nd a 0.061 ± 0.005 b	0.037 ± 0.032 a nd a 0.015 ± 0.006 a	A
al4	1118	2-Cyclohexen-1-ol (822-67-3)	SPME SD SE	nd a nd a 0.018 ± 0.012 a	nd a nd a 0.013 ± 0.012 a	nd a nd a nd a	A
al5	1124	2-Cyclohexen-2-ol	SPME SD SE	nd a nd a 0.020 ± 0.011 a	nd a nd a 0.018 ± 0.016 a	nd a nd a nd a	C
al6	1154	1-Octen-3-ol (3391-86-4)	SPME SD SE	0.828 ± 0.174 c 0.361 ± 0.039 c 0.482 ± 0.045 c	0.490 ± 0.075 b 0.267 ± 0.050 b 0.247 ± 0.027 b	0.078 ± 0.001 a 0.008 ± 0.007 a nd a	A
al7	1427	1-Decanol (112-30-1)	SPME SD SE	nd a 0.274 ± 0.039 b nd a	nd a nd a nd a	nd a nd a nd a	C
al8	1499	2,4-Decadien-1-ol (14507-02-9)	SPME SD SE	nd a nd a nd a	nd a nd a nd a	nd a 0.126 ± 0.022 b 0.159 ± 0.031 b	C
al9	1630	1-Tetradecanol (112-72-1)	SPME SD SE	nd a nd a 0.071 ± 0.010 b	nd a nd a nd a	nd a nd a 0.073 ± 0.012 b	C
**Carbonyls**							
c1	<800	1-Penten-3-one (1629-58-9)	SPME SD SE	0.072 ± 0.008 b nd a 0.036 ± 0.004 c	0.074 ± 0.017 b nd a 0.027 ± 0.004 b	nd a nd a nd a	C
c2	902	Hexanal (66-25-1)	SPME SD SE	0.293 ± 0.042 b 0.046 ± 0.021 b nd a	0.346 ± 0.020 b 0.030 ± 0.015 ab 0.062 ± 0.012 b	0.041 ± 0.013 a 0.004 ± 0.004 a nd a	A
c3	911	2-Methyl-2-pentenal (623-36-9)	SPME SD SE	0.040 ± 0.003 b nd a 1.055 ± 0.122 b	0.117 ± 0.015 c nd a 1.557 ± 0.080 c	nd a nd a nd a	C
c4	983	2-Hexenal (505-57-7)	SPME SD SE	0.757 ± 0.197 b 0.134 ± 0.055 b 0.138 ± 0.005 b	1.907 ± 0.326 c 0.189 ± 0.072 b 0.159 ± 0.020 b	0.159 ± 0.020 b 0.016 ± 0.004 a 0.025 ± 0.018 a	A
c5	1056	Hexa-2,4-dienal (80466-34-8)	SPME SD SE	nd a nd a nd a	0.160 ± 0.024 b nd a nd a	nd a nd a nd a	A
c6	1201	2-Octenal (2363-89-5)	SPME SD SE	0.300 ± 0.113 a 0.040 ± 0.002 a nd a	0.397 ± 0.102 a 0.046 ± 0.008 a nd a	1.724 ± 0.316 b 0.042 ± 0.005 a nd a	A
c7	1309	Decanal (112-31-2)	SPME SD SE	nd a 0.060 ± 0.004 b 0.065 ± 0.056 a	nd a nd a nd a	nd a nd a nd a	A
c8	1752	3,4-Dimethyl-2-cyclohexen-1-carboxaldehyde	SPME SD SE	nd a ^5^ nd a nd a	nd ^6^ a nd a nd a	0.475 ± 0.068 b nd a nd a	C
**Esters**							
e1	1171	1-Octenyl acetate (77149-68-9) ^7^	SPME SD SE	nd a nd a nd a	0.105 ± 0.014 c 0.259 ± 0.046 b 0.042 ± 0.004 b	0.038 ± 0.008 b nd a nd a	C
e2	1494	Methyl-2,4-Decadienoate (7328-33-8)	SPME SD SE	nd a nd a nd a	nd a nd a nd a	nd a 0.069 ± 0.010 b 0.073 ± 0.012 b	C
e3	1705	3-Hexenyl benzoate (72200-74-9)	SPME SD SE	nd a 0.054 ± 0.003 b nd a	nd a nd a nd a	nd a nd a nd a	C
**Hydrocarbons**							
h1	<800	Cyclohexene (110-83-8)	SPME SD SE	nd a 0.131 ± 0.003 b 1.349 ± 0.213 a	nd a 0.139 ± 0.032 b 1.878 ± 0.252 b	nd a 0.044 ± 0.006 a 1.174 ± 0.223 a	C
h2	811	2-Methyl-2-heptane	SPME SD SE	nd a nd a 0.014 ± 0.001 a	nd a nd a 0.012 ± 0.002 a	nd a nd a 0.013 ± 0.001 a	C
h3	874	1,2,4,4-Tetramethyl-cyclopentene (65378-76-9)	SPME SD SE	0.012 ± 0.002 b nd a nd a	nd a nd a nd a	0.041 ± 0.002 c nd a nd a	C
h4	899	2,3-Dimethyl -1-pentene (3404-72-6)	SPME SD SE	0.015 ± 0.002 a nd a 0.012 ± 0.001 b	0.051 ± 0.014 b nd a nd a	nd a nd a nd a	C
h5	974	Methyl cyclohexane (108-87-2)	SPME SD SE	nd a nd a nd a	0.063 ± 0.009 b nd a nd a	nd a nd a nd a	C
h6	1027	3,7,7-Trimethyl-1,3,5-Cycloheptatriene (3479-89-8)	SPME SD SE	0.371 ± 0.019 b 0.589 ± 0.023 b 0.436 ± 0.067 b	nd a nd a nd a	nd a nd a nd a	C
h7	1041	2-Methyl-1,3-Pentadiene (1118-58-7)	SPME SD SE	nd a nd a nd a	nd a nd a 0.058 ± 0.006 b	nd a nd a nd a	C
h8	1167	Ethyl cyclohexane (1678-91-7)	SPME SD SE	nd a nd a 0.036 ± 0.006 b	0.055 ± 0.020 b nd a 0.048 ± 0.006 c	nd a nd a nd a	C
h9	1200	1,4,8-*p*-Menthatriene	SPME SD SE	nd a 0.038 ± 0.005 c 0.021 ± 0.004 b	nd a nd a nd a	nd a 0.008 ± 0.001 b nd a	C
h10	1221	1,3,5-Undecatriene (16356-11-9)	SPME SD SE	nd a nd a 0.032 ± 0.002 b	nd a nd a nd a	0.151 ± 0.015 b 0.022 ± 0.004 b 0.056 ± 0.004 c	C
h11	1423	Cyclodecene (3618-12-0)	SPME SD SE	nd a 0.103 ± 0.013 b 0.307 ± 0.037 b	nd a nd a nd a	nd a nd a nd a	C
h12	1622	1,3,5-Trimethyl cyclohexane (1839-63-0)	SPME SD SE	nd a nd a nd a	nd a 0.077 ± 0.015 b nd a	nd a nd a nd a	C
h13	1627	1-Decene (872-05-9)	SPME SD SE	nd a nd a nd a	nd a nd a nd a	nd a 0.054 ± 0.008 b nd a	C
**Benzenes and Benzene Derivatives**							
b1	1107	Benzaldehyde (100-52-7)	SPME SD SE	0.081 ± 0.017 a nd a nd a	0.104 ± 0.031 a nd a nd a	2.058 ± 0.143 b 0.008 ± 0.001 b nd a	A
b2	1307	Benzenmethanol (100-51-6) ^7^	SPME SD SE	0.239 ± 0.011 a ^5^ nd ^6^ a 0.084 ± 0.017 b	0.433 ± 0.079 c nd a nd a	0.336 ± 0.071 ab nd a nd a	C
b3	1401	Anethole	SPME SD SE	nd a nd a nd a	0.125 ± 0.004 b 0.781 ± 0.073 b 0.301 ± 0.042 b	nd a nd a nd a	C
b4	1587	Cuparene (16982-00-6)	SPME SD SE	0.103 ± 0.028 a nd a 0.436 ± 0.067 b	0.054 ± 0.008 a 0.071 ± 0.012 b 0.170 ± 0.011 a	0.706 ± 0.279 b 0.082 ± 0.030 b 0.142 ± 0.022 a	B
b5	1963	Benzyl benzoate (120-51-4)	SPME SD SE	nd a 0.099 ± 0.010 b 0.300 ± 0.027 b	nd a nd a nd a	nd a nd a nd a	C

^1^ Retention indices (RI) were determined using *n*-paraffins C_7_–C_22_ as external standards on Cyclodex-B column; ^2^ All volatile compounds, positively identified by matching mass spectrum and retention index with those of an authentic standard, are listed by the order of their RI in a chemical class; ^3^ Volatile compounds were calculated with the relative peak ratio of their peak areas to that of internal standard (*n* = 3) ± standard deviation; ^4^ Identification of volatiles was performed requiring the following criteria: A, mass spectrum and retention index were consistent with those of an authentic standard (positive identification); B, mass spectrum and retention index were consistent with those of literatures [6,20,25]; C^,^mass spectrum was consistent with that of Wiley 7n spectral database (Agilent Technologies) or by manual interpretation (tentative identification); ^5^ Difference letters mean significant differences (*p* < 0.05) between three different needle samples according to three different cultivar species or extraction methods by Duncan’s multiple range test; ^6^ nd = not detected; ^7^ CAS Registry number.

**Table 2:** Monoterpenes (monoterpene hydrocarbons and oxygenated monoterpenes) of three different cultivars based on different extraction methods.

Relative Peak Area (Mean ± SD) ^3^							
No.	RI ^1^	Volatile Compounds ^2^	Extraction Methods	Cultivar Species			ID ^4^
				CP	CO	TO	
**Monoterpenes (C_10_H_16_) Monoterpene Hydrocarbons**							
mh1	951	2-Bornene (464-17-5) ^7^	SPME SD SE	nd a ^5^ nd a nd a	nd ^6^ a nd a nd a	nd a 0.025 ± 0.001 b 0.034 ± 0.003 b	C
mh2	958	α-Thujene (2867-05-2)	SPME SD SE	0.157 ± 0.010 a 0.355 ± 0.033 a 0.775 ± 0.076 b	1.783 ± 0.555 b 1.239 ± 0.325 b 0.339 ± 0.030 a	1.806 ± 0.219 b 0.221 ± 0.028 a 0.421 ± 0.033 a	B
mh3	992	(−)-α-Pinene (7785-26-4)	SPME SD SE	3.579 ± 0.342 b 3.497 ± 0.460 c 3.412 ± 0.491 c	0.047 ± 0.003 a 0.102 ± 0.025 a 0.026 ± 0.002 a	9.687 ± 1.133 c 1.624 ± 0.314 b 2.230 ± 0.283 b	A
mh4	994	(+)-α-Pinene (80-56-8)	SPME SD SE	51.469 ± 7.022 b 54.083 ± 3.942 b 52.566 ± 9.930 b	0.932 ± 0.160 a 3.265 ± 1.014 a 0.890 ± 0.058 a	58.154 ± 4.405 b 8.200 ± 1.769 a 11.634 ± 1.545 a	A
mh5	1013	Tricyclene (508-32-7)	SPME SD SE	0.978 ± 0.076 c 0.880 ± 0.063 c 0.119 ± 0.005 c	0.052 ± 0.012 a 0.229 ± 0.061 b 0.051 ± 0.009 a	0.498 ± 0.018 b 0.051 ± 0.005 a 0.079 ± 0.010 b	B
mh6	1017	β-Myrcene (123-35-3)	SPME SD SE	22.647 ± 1.413 c 34.756 ± 3.354 b 26.138 ± 4.473 b	2.197 ± 0.581 a 6.874 ± 2.315 a 1.837 ± 0.107 a	10.973 ± 2.083 b 2.393 ± 0.441 a 3.336 ± 0.581 a	A
mh7	1022	(+ or −)-Sabinene (3387-41-5)	SPME SD SE	nd a nd a nd a	5.309 ± 1.207 b 18.172 ± 4.703 b 6.167 ± 0.711 c	3.959 ± 0.301 b 0.657 ± 0.099 a 1.446 ± 0.155 b	A
mh8	1023	α-Fenchene (471-84-1) ^7^	SPME SD SE	nd a ^5^ nd a nd a	nd ^6^ a nd a nd a	2.027 ± 0.245 b nd a nd a	B
mh9	1027	Camphene (79-92-5)	SPME SD SE	0.926 ± 0.050 c 1.202 ± 0.071 b 0.579 ± 0.231 b	0.249 ± 0.042 b 0.766 ± 0.682 ab 0.239 ± 0.030 a	nd a 0.024 ± 0.002 a 0.039 ± 0.007 a	A
mh10	1030	(+ or −)-Sabinene (3387-41-5)	SPME SD SE	nd a nd a nd a	nd a nd a nd a	2.750 ± 0.364 b 0.272 ± 0.053 b 0.647 ± 0.067 b	A
mh11	1039	4-Carene	SPME SD SE	nd a nd a nd a	nd a nd a nd a	0.794 ± 0.089 b 0.078 ± 0.007 b 0.115 ± 0.014 b	B
mh12	1045	β-Pinene (127-91-3)	SPME SD SE	nd a nd a nd a	0.146 ± 0.025 b 0.449 ± 0.195 b 0.091 ± 0.011 b	nd a nd a nd a	A
mh13	1048	α-Phellandrene(99-83-2)	SPME SD SE	nd a nd a 24.974 ± 5.025 c	nd a 0.210 ± 0.065 b nd a	nd a nd a 13.826 ± 1.316 b	A
mh14	1051	δ-3-Carene (13466-78-9)	SPME SD SE	20.460 ± 3.370 b 24.721 ± 3.762 c nd a	nd a nd a nd a	37.422 ± 6.341 c nd a 10.141 ± 1.287 b	B
mh15	1060	α-Terpinene (99-86-5)	SPME SD SE	0.149 ± 0.008 a 0.384 ± 0.037 a 0.495 ± 0.066 b	0.633 ± 0.102 b 3.036 ± 0.479 b 0.055 ± 0.008 a	0.808 ± 0.050 c 0.152 ± 0.013 a 0.070 ± 0.004 a	A
mh16	1069	*m*-Mentha-6,8-diene	SPME SD SE	0.216 ± 0.020 b 0.262 ± 0.041 c 0.207 ± 0.035 c	nd a nd a nd a	0.386 ± 0.039 c 0.086 ± 0.002 b 0.096 ± 0.011 b	C
mh17	1075	(-)-Limonene (5989-54-8)	SPME SD SE	0.866 ± 0.088 b 1.089 ± 0.109 b 0.866 ± 0.111 c	0.129 ± 0.054 a 0.225 ± 0.027 a 0.037 ± 0.005 a	1.523 ± 0.189 c 0.246 ± 0.011 a 0.266 ± 0.033 b	A
mh18	1078	(+)-Limonene (5989-54-8)	SPME SD SE	2.577 ± 0.236 a 3.937 ± 0.377 b 3.324 ± 0.620 b	3.006 ± 0.488 a 13.712 ± 2.238 c 2.890 ± 0.267 b	2.484 ± 0.283 a 0.395 ± 0.031 a 0.495 ± 0.076 a	A
mh19	1092	(-)-β-Phellandrene (555-10-2)	SPME SD SE	0.245 ± 0.033 a 0.388 ± 0.055 a 0.340 ± 0.044 a	0.042 ± 0.007 a 0.112 ± 0.058 a 0.017 ± 0.004 a	22.727 ± 4.501 b 4.350 ± 0.658 b 6.563 ± 0.676 b	B
mh20	1095	(+)-β-Phellandrene (555-10-2)	SPME SD SE	nd a 0.119 ± 0.018 a 0.141 ± 0.024 c	nd a 0.161 ± 0.140 a nd a	nd a 0.073 ± 0.005a 0.066 ± 0.003 b	B
mh21	1105	γ-Terpinene (99-85-4)	SPME SD SE	0.166 ± 0.019 a 0.492 ± 0.051 a 0.427 ± 0.037 b	1.797 ± 0.346 c 9.184 ± 1.787 b 1.251 ± 0.110 c	0.849 ± 0.096 b 0.219 ± 0.005 a 0.111 ± 0.006 a	A
mh22	1128	α-Terpinolene	SPME SD SE	1.746 ± 0.370 a 3.797 ± 0.538 b 2.940 ± 0.514 b	1.288 ± 1.022 a 3.048 ± 0.582 b 0.364 ± 0.044 a	5.420 ± 0.812 b 2.085 ± 0.145 a 2.565 ± 0.206 b	B
**Monoterpenes (C_10_H_16_) Oxygenated Monoterpenes**							
mo1	1122	1,8-Cineole (470-82-6)	SPME SD SE	nd a nd a 0.005 ± 0.004 a	nd a nd a nd a	nd a 0.005 ± 0.004 a 0.018 ± 0.002 b	A
mo2	1214	α-Fenchone	SPME SD SE	nd a nd a 0.013 ± 0.011 a	nd a nd a nd a	nd a nd a nd a	C
mo3	1215	Linalool oxide (60047-17-8)	SPME SD SE	nd a nd a 0.013 ± 0.001 b	nd a nd a nd a	nd a nd a nd a	A
mo4	1225	Sabinene hydrate (546-79-2)^7^	SPME SD SE	2.332 ± 0.819 b ^5^ 0.065 ± 0.012 b 0.201 ± 0.025 c	0.150 ± 0.023 a 0.055 ± 0.028 b 0.149 ± 0.014 b	nd ^6^ a nd a nd a	A
mo5	1236	Linalool (78-70-6)	SPME SD SE	nd a nd a nd a	0.117 ± 0.006 b 0.388 ± 0.047 b nd a	nd a nd a nd a	A
mo6	1247	Limonene oxide (1195-92-2)	SPME SD SE	nd a nd a 0.079 ± 0.004 b	nd a nd a nd a	nd a nd a nd a	A
mo7	1248	Phellandral (21391-98-0)	SPME SD SE	nd a nd a nd a	nd a nd a nd a	0.099 ± 0.011 b nd a nd a	C
mo8	1257	Campholene aldehyde (4501-58-0)	SPME SD SE	0.128 ± 0.007 b 0.020 ± 0.002 b 0.036 ± 0.031 ab	nd a nd a nd a	0.492 ± 0.025 c 0.010 ± 0.009ab 0.049 ± 0.007 b	C
mo9	1289	Carvacrol methyl ester	SPME SD SE	nd a nd a 0.019 ± 0.003 b	nd a nd a nd a	nd a nd a nd a	C
mo10	1290	*p*-Menth-2-en-1-ol	SPME SD SE	nd a nd a nd a	nd a 0.593 ± 0.096 b nd a	nd a 0.075 ± 0.002 a nd a	C
mo11	1318	Camphor (76-22-2)	SPME SD SE	0.054 ± 0.005 b 0.043 ± 0.001 b 0.070 ± 0.020 b	0.026 ± 0.004 a nd a nd a	0.025 ± 0.003 a nd a nd a	B
mo12	1320	Linalyl acetate (115-95-7)	SPME SD SE	0.033 ± 0.004 b 0.021 ± 0.001 a 0.080 ± 0.021 b	0.082 ± 0.007 c 0.368 ± 0.053 b 0.235 ± 0.025 c	nd a nd a nd a	A
mo13	1324	1-Terpineol (586-82-3)	SPME SD SE	nd a nd a nd a	nd a nd a 0.383 ± 0.048 b	nd a 0.043 ± 0.002 b 0.343 ± 0.042 b	C
mo14	1325	Fenchyl alcohol	SPME SD SE	nd a nd a nd a	0.021 ± 0.006 b nd a nd a	nd a nd a nd a	C
mo15	1326	(−)-4-Terpineol (20126-76-5)	SPME SD SE	0.120 ± 0.015 b 0.362 ± 0.027 a 0.218 ± 0.005 c	0.133 ± 0.016 b 10.820 ± 1.034 b nd a	0.058 ± 0.009 a 0.212 ± 0.028 a 0.078 ± 0.019 b	A
mo16	1327	(+)-4-Terpineol	SPME SD SE	nd a 0.463 ± 0.068 c 0.323 ± 0.040 c	0.028 ± 0.008 b nd a nd a	0.215 ± 0.020 c 0.231 ± 0.023 b 0.090 ± 0.007 b	A
mo17	1328	Pinocarveol (5947-36-4)	SPME SD SE	0.060 ± 0.005 b 0.038 ± 0.003 b nd a	nd a nd a nd a	nd a nd a nd a	C
mo18	1331	Pinocarvone	SPME SD SE	0.116 ± 0.009 b nd a nd a	nd a nd a nd a	0.149 ± 0.010 c nd a nd a	C
mo19	1339	α-Phellandren-8-ol (1686-20-0)	SPME SD SE	nd a nd a nd a	nd a nd a nd a	nd a 0.057 ± 0.008 b nd a	C
mo20	1347	1,8-menthadien-4-ol	SPME SD SE	nd a 0.079 ± 0.005 b 0.080 ± 0.011 b	nd a 0.183 ± 0.030 c nd a	nd a nd a nd a	C
mo21	1352	Camphene hydrate (465-31-6)	SPME SD SE	0.074 ± 0.004 b nd a nd a	0.064 ± 0.026 b nd a nd a	nd a nd a nd a	C
mo22	1367	α-Terpineol (98-55-5)	SPME SD SE	nd a 0.064 ± 0.009 a nd a	nd a 1.294 ± 0.308 b nd a	nd a 0.116 ± 0.015 a nd a	A
mo23	1375	(−)-Bornyl acetate (5655-61-8) ^7^	SPME SD SE	12.435 ± 1.319 b ^5^ 15.101 ± 1.983 b 14.794 ± 1.912 c	2.285 ± 0.163 a 20.935 ± 3.605 c 2.854 ± 0.232 b	1.032 ± 0.107 a 0.177 ± 0.025 a 0.434 ± 0.059 a	C
mo24	1384	(+)-Bornyl acetate	SPME SD SE	nd ^6^ a nd a 0.503 ± 0.056 c	0.151 ± 0.008 b 2.943 ± 0.226 b 0.337 ± 0.047 b	nd a 0.051 ± 0.008 a nd a	A
mo25	1405	Borneol	SPME SD SE	0.356 ± 0.047 c 0.553 ± 0.042 b 0.583 ± 0.055 b	0.155 ± 0.023 a nd a nd a	0.231 ± 0.012 b nd a nd a	A
mo26	1422	Geraniol (106-24-1)	SPME SD SE	nd a nd a nd a	nd a 0.110 ± 0.014 b nd a	nd a nd a nd a	C
mo27	1435	Terpinenyl acetate (80-26-2)	SPME SD SE	1.506 ± 0.377 a 3.708 ± 0.536 a 5.079 ± 0.498 b	2.268 ± 0.226 a 38.222 ± 5.323 b 7.489 ± 0.371 c	7.985 ± 1.365 b 3.132 ± 0.463 a 3.981 ± 0.625 a	C
mo28	1472	Geranyl acetate (105-87-3)	SPME SD SE	nd a 0.073 ± 0.014 b nd a	nd a nd a nd a	nd a nd a nd a	C
mo29	1538	Methyl eugenol (93-15-2)	SPME SD SE	nd a 0.029 ± 0.004 b nd a	nd a nd a nd a	nd a nd a nd a	B
mo30	2129	γ-Hydroxyisoeugenol (458-35-5)	SPME SD SE	nd a nd a 0.247 ± 0.021 b	nd a nd a nd a	nd a nd a 0.469 ± 0.047 c	C

^1^ Retention indices (RI) were determined using *n*-paraffins C_7_–C_22_ as external standards on Cyclodex-B column; ^2^ All volatile compounds, positively identified by matching mass spectrum and retention index with those of an authentic standard, are listed by the order of their RI in a chemical class; ^3^ Volatile compounds were calculated with the relative peak ratio of their peak areas to that of internal standard (*n* = 3) ± standard deviation; ^4^ Identification of volatiles was performed requiring the following criteria: A, mass spectrum and retention index were consistent with those of an authentic standard (positive identification); B, mass spectrum and retention index were consistent with those of literatures [6,20,25]; C^,^mass spectrum was consistent with that of Wiley 7n spectral database (Agilent Technologies) or by manual interpretation (tentative identification); ^5^ Difference letters mean significant differences (*p* < 0.05) between three different needle samples according to three different cultivar species or extraction methods by Duncan’s multiple range test; ^6^ nd = not detected; ^7^ CAS Registry number.

**Table 3:** Sesquiterpenes (Sesquiterpene hydrocarbons and oxygenated sesquiterpenes) and diterpenes of three different cultivars based on different extraction methods.

Relative Peak Area (Mean ± SD) ^3^							
No.	RI^1^	Volatile Compounds ^2^	Extraction Methods	Cultivar Species			ID ^4^
				CP	CO	TO	
**Sesquiterpenes (C_15_H_24_) Sesquiterpene Hydrocarbons**							
sh1	1367	α-Longipinene (5989-08-2) ^7^	SPME SD SE	0.077 ± 0.015 b ^5^ nd a 0.048 ± 0.007 c	nd ^6^ a nd a 0.013 ± 0.001 b	nd a nd a nd a	A
sh2	1392	*di*-*epi*-α-Cedrene (1) (50894-66-1)	SPME SD SE	nd a nd a nd a	nd a nd a nd a	0.480 ± 0.035 b nd a nd a	C
sh3	1403	α-Cedrene (469-61-4)	SPME SD SE	nd a nd a nd a	nd a nd a nd a	0.644 ± 0.067 b nd a 0.047 ± 0.009 b	A
sh4	1409	β-Bourbonene (5208-59-3) ^7^	SPME SD SE	nd a ^5^ nd a nd a	nd ^6^ a nd a nd a	1.412 ± 0.167 b 0.043 ± 0.002 b 0.343 ± 0.042 b	C
sh5	1415	α-Bisabolene	SPME SD SE	nd a nd a nd a	nd a nd a nd a	0.647 ± 0.034 b nd a nd a	C
sh6	1427	β-Elemene	SPME SD SE	0.033 ± 0.006 a nd a nd a	0.058 ± 0.003 a 0.138 ± 0.098 b 0.248 ± 0.030 b	0.705 ± 0.046 b 0.221 ± 0.057 b 0.708 ± 0.147 c	C
sh7	1444	Aromadendrene (109119-91-7)	SPME SD SE	nd a 0.089 ± 0.010 b nd a	nd a nd a nd a	nd a nd a nd a	A
sh8	1445	Longifolene (475-20-7)	SPME SD SE	1.571 ± 0.261 b nd a 1.569 ± 0.121 b	0.067 ± 0.006 a nd a nd a	10.277 ± 1.687 b 0.618 ± 0.067 b nd a	A
sh9	1447	β-Cedrene (546-28-1)	SPME SD SE	0.423 ± 0.038 a nd a nd a	0.067 ± 0.006 a nd a nd a	10.277 ± 1.687 b 0.618 ± 0.067 b nd a	A
sh10	1469	(+ or -)-γ-Muurolene (30021-74-0)	SPME SD SE	0.286 ± 0.015 b nd a 0.370 ± 0.041 a	nd a nd a 0.229 ± 0.025 a	2.200 ± 0.206 c 0.134 ± 0.013 b 1.775 ± 0.303 b	C
sh11	1471	Thujopsene (470-40-6)	SPME SD SE	nd a nd a nd a	0.605 ± 0.042 b 6.492 ± 1.235 b 1.804 ± 0.048 c	2.313 ± 0.296 c 0.771 ± 0.171 a 0.911 ± 0.104 b	C
sh12	1479	(+ or -)-γ-Muurolene (30021-74-0)	SPME SD SE	0.101 ± 0.016 a nd a 0.388 ± 0.036 a	0.089 ± 0.004 a 1.713 ± 0.265 c 0.301 ± 0.034 a	8.130 ± 1.202 b 0.571 ± 0.084 b 1.126 ± 0.180 b	C
sh13	1492	(−)-Caryophyllene (87-44-5)	SPME SD SE	1.049 ± 0.295 a 0.103 ± 0.015 a 2.333 ± 0.330 a	0.178 ± 0.007 a 0.035 ± 0.016 a 0.632 ± 0.095 a	21.297 ± 3.296 b 2.266 ± 0.305 b 9.489 ± 1.667 b	A
sh14	1492	(+)-Caryophyllene (87-44-5)	SPME SD SE	0.227 ± 0.047 b nd a 0.867 ± 0.133 b	0.049 ± 0.014a 0.035 ± 0.021 b 0.420 ± 0.004 a	0.822 ± 0.123 c 0.106 ± 0.016 c 0.321 ± 0.058 a	A
sh15	1503	α-Copaene (3856-25-5)	SPME SD SE	nd a 0.064 ± 0.011 b 0.296 ± 0.044b	nd a nd a nd a	nd a 0.156 ± 0.021 c 0.420 ± 0.069 c	A
sh16	1504	(+ or −)-γ-Curcumene	SPME SD SE	0.133 ± 0.023 a 0.033 ± 0.005 a nd a	nd a nd a nd a	1.192 ± 0.204 b 0.203 ± 0.041 b nd a	C
sh17	1506	Bicyclo Sesquiphellandrene	SPME SD SE	nd a nd a nd a	nd a 3.216 ± 0.574 b 0.537 ± 0.044 b	nd a nd a nd a	C
sh18	1507	*di*-*epi*-α-Cedrene (2) (50894-66-1)	SPME SD SE	0.201 ± 0.031 a nd a 0.388 ± 0.056 b	nd a nd a nd a	2.198 ± 0.277 b nd a 0.415 ± 0.077 b	C
sh19	1514	(+ or − )-γ-Curcumene	SPME SD SE	0.421 ± 0.110 a nd a nd a	nd a nd a nd a	3.716 ± 0.796 b nd a nd a	C
sh20	1515	α-Humulene (6753-98-6)	SPME SD SE	nd a nd a nd a	0.029 ± 0.001 a nd a 0.141 ± 0.027 a	11.356 ± 0.823 b 3.624 ± 0.538 b 11.040 ± 2.103 b	A
sh21	1533	β-Chamigrene (18431-82-8)	SPME SD SE	nd a nd a nd a	nd a 0.082 ± 0.026 b 0.394 ± 0.046 b	nd a nd a nd a	C
sh22	1534	α-Curcumene (644-30-4)	SPME SD SE	0.331 ± 0.084 a 0.033 ± 0.006 a 1.094 ± 0.198 b	nd a nd a nd a	5.230 ± 1.093 b 0.488 ± 0.042 b 0.902 ± 0.139 b	C
sh23	1538	Isoledene (95910-36-4) ^7^	SPME SD SE	nd a ^5^ nd a nd a	nd ^6^ a 1.693 ± 0.431 b nd a	nd a nd a nd a	C
sh24	1539	Germacrene-D (23986-74-5)	SPME SD SE	nd a nd a nd a	0.131 ± 0.001 a nd a 0.926 ± 0.111 a	5.170 ± 0.610 b 1.345 ± 0.223 b 6.272 ± 1.209 b	C
sh25	1541	β-Humulene (116-04-1)	SPME SD SE	0.543 ± 0.154 b nd a 1.232 ± 0.130 b	nd a nd a nd a	nd a nd a nd a	C
sh26	1556	α-Chamigrene (19912-83-5)	SPME SD SE	nd a nd a nd a	nd a 0.542 ± 0.155 b nd a	0.647 ± 0.034 b nd a nd a	C
sh27	1559	β-Himachalene (1) (1461-03-6)	SPME SD SE	nd a nd a nd a	0.052 ± 0.005 b 2.749 ± 0.573 b 0.368 ± 0.033 c	0.116 ± 0.001 c 0.131 ± 0.010 a 0.172 ± 0.014 b	C
sh28	1564	γ-Bisabolene	SPME SD SE	0.051 ± 0.012 a 0.005 ± 0.005 a 0.408 ± 0.064 b	0.021 ± 0.009 a nd a 0.231 ± 0.028 a	0.200 ± 0.024 b 0.117 ± 0.011 b 0.312 ± 0.056 ab	B
sh29	1569	α-Amorphene (20085-19-2)	SPME SD SE	nd a nd a nd a	nd a nd a 0.070 ± 0.012 b	nd a 0.137 ± 0.025 b 0.358 ± 0.058 c	C
sh30	1571	γ-Cadinene (39029-41-9)	SPME SD SE	nd a nd a nd a	0.012 ± 0.002 a 1.464 ± 0.397 c nd a	0.530 ± 0.045 b 0.531 ± 0.120 b nd a	C
sh31	1574	β-Sesquiphellandrene (20307-83-9)	SPME SD SE	0.069 ± 0.021 b nd a 0.531 ± 0.072 b	nd a nd a nd a	nd a nd a 0.785 ± 0.149 c	C
sh32	1575	δ-Cadinene (483-76-1)	SPME SD SE	nd a nd a 0.082 ± 0.008 b	0.024 ± 0.005 a nd a nd a	0.764 ± 0.087 b nd a nd a	C
sh33	1591	β-Himachalene (2) (1461-03-6)	SPME SD SE	nd a nd a nd a	nd a 1.240 ± 0.189 a 0.515 ± 0.061 c	nd a 0.124 ± 0.024 b 0.196 ± 0.029 b	C
sh34	1616	Germacrene-B (15423-57-1)	SPME SD SE	nd a nd a 0.192 ± 0.041 a	0.178 ± 0.007 a 0.092 ± 0.015 b 0.238 ± 0.031 a	0.187 ± 0.025 b 0.220 ± 0.042 c 0.703 ± 0.118 b	C
sh35	1660	Calarene (17334-55-3)	SPME SD SE	nd a nd a nd a	nd a nd a 1.390 ± 0.145 b	nd a nd a nd a	C
sh36	1786	β-Patchoulene (514-51-2)	SPME SD SE	nd a nd a nd a	nd a 0.151 ± 0.031 b nd a	nd a nd a nd a	C
sh37	1789	β-Panasinsene	SPME SD SE	nd a nd a nd a	nd a 0.200 ± 0.048 b nd a	nd a nd a nd a	C
sh38	1795	Bicyclogermacrene	SPME SD SE	nd a nd a nd a	nd a nd a nd a	nd a 0.311 ± 0.073 b nd a	C
sh39	1829	β-Selinene (17066-67-0)	SPME SD SE	nd a 0.031 ± 0.004 b 0.210 ± 0.024 b	nd a nd a nd a	nd a nd a nd a	C
sh40	2036	Eudesma-4(14),11-diene (17066-67-0)	SPME SD SE	nd a nd a 22.291 ± 3.688 b	nd a nd a nd a	nd a nd a nd a	C
**Sesquiterpenes (C_15_H_24_) Oxygenated Sesquiterpenes**							
so1	1692	Nerolidol (142-50-7) ^7^	SPME SD SE	nd a ^5^ 0.210 ± 0.034 b 1.507 ± 0.219 b	nd ^6^ a 0.204 ± 0.053 b 0.103 ± 0.034 a	nd a 0.094 ± 0.010 a 0.143 ± 0.004 a	A
so2	1702	Elemol (639-99-6)	SPME SD SE	nd a nd a nd a	nd a 28.098 ± 4.173 b 14.885 ± 1.305 c	nd a 0.773 ± 0.419 a 1.880 ± 0.361 b	C
so3	1713	Germacrene-D-4-ol (198991-79-6)	SPME SD SE	nd a nd a 0.164 ± 0.027 a	nd a nd a 0.416 ± 0.066 a	nd a 0.063 ± 0.011 b 1.715 ± 0.328 b	C
so4	1728	Caryophyllene oxide (1139-30-6)	SPME SD SE	0.067 ± 0.023 a 0.112 ± 0.012 a 0.704 ± 0.071 c	nd a nd a nd a	0.977 ± 0.239 b 0.546 ± 0.135 b 0.336 ± 0.040 b	A
so5	1730	Spathulanol	SPME SD SE	nd a nd a nd a	nd a nd a nd a	nd a 3.415 ± 1.151 b 6.346 ± 0.993 b	C
so6	1731	Longipinanol	SPME SD SE	nd a nd a nd a	nd a 0.583 ± 0.101 b nd a	nd a nd a nd a	C
so7	1745	α-Cadinol (481-34-5)	SPME SD SE	nd a nd a nd a	nd a nd a nd a	nd a 0.232 ± 0.060 b nd a	C
so8	1762	Widdrol (6892-80-4)	SPME SD SE	nd a nd a nd a	nd a 2.637 ± 0.496 b nd a	nd a nd a nd a	C
so9	1775	α-Cedrol (77-53-2)	SPME SD SE	nd a nd a 0.181 ± 0.017 b	nd a nd a nd a	nd a nd a 0.226 ± 0.038 b	C
so10	1781	γ-Eudesmol (1209-71-8)	SPME SD SE	nd a nd a nd a	nd a 7.999 ± 1.427 b 0.143 ± 0.013 b	nd a nd a nd a	C
so11	1795	β-Bisabolol (15352-77-9)	SPME SD SE	nd a 0.058 ± 0.005 b 0.238 ± 0.015 a	nd a nd a nd a	nd a nd a 0.403 ± 0.064 b	C
so12	1806	Gossonorol (92691-77-5)	SPME SD SE	nd a nd a 0.299 ± 0.033 b	nd a nd a nd a	nd a nd a nd a	C
so13	1809	α-Eudesmol (473-16-5)	SPME SD SE	nd a 0.058 ± 0.004 a 0.238 ± 0.015 a	nd a 6.469 ± 1.588 b 1.477 ± 0.318 b	nd a 0.297 ± 0.140 a 0.213 ± 0.031 a	C
so14	1820	β-Eudesmol (473-15-4)	SPME SD SE	nd a 0.043 ± 0.006 a 0.258 ± 0.040 a	nd a 5.794 ± 1.121 b 0.760 ± 0.113 b	nd a 0.598 ± 0.351 a 0.325 ± 0.057 a	C
so15	1825	α-Bisabolol (515-69-5)	SPME SD SE	nd a 0.088 ± 0.006 a 0.761 ± 0.117 c	nd a 0.491 ± 0.128 b 0.322 ± 0.044 b	nd a nd a nd a	C
so16	1867	Cedryl acetate (77-54-3)	SPME SD SE	nd a nd a nd a	nd a nd a nd a	0.320 ± 0.044 b 0.945 ± 0.455 b 1.811 ± 0.334 b	C
**Diterpenes (C_20_H_32_) Diterpene Hydrocarbons**							
dh1	1786	Rimuen (1686-67-5)	SPME SD SE	1.667 ± 1.118 b 1.422 ± 0.208 b 4.420 ± 0.169 b	1.493 ± 0.234 b 4.406 ± 0.785 c 9.946 ± 0.203 c	nd a nd a nd a	C
dh2	1789	*Ent*-pimara-8,15-diene	SPME SD SE	nd a 0.040 ± 0.036 a nd a	nd a nd a nd a	nd a nd a 0.168 ± 0.015 b	C
dh3	1795	Stachene (3564-54-3)	SPME SD SE	4.325 ± 2.203 a 5.044 ± 0.609 a 15.412 ± 1.025 b	9.424 ± 3.081 b 25.773 ± 4.441 b 60.483 ± 5.198 c	nd a nd a nd a	C
dh4	1829	*Ent*-pimara-8(14),15-diene	SPME SD SE	nd a ^5^ 0.339 ± 0.024 b 1.312 ± 0.191 b	nd ^6^ a 1.385 ± 0.041 c 3.329 ± 0.258 c	nd a nd a nd a	C
dh5	2036	Kaur-16-ene (562-28-7) ^7^	SPME SD SE	nd a nd a nd a	nd a nd a 0.770 ± 0.093 b	nd a nd a nd a	C
dh6	1692	Isopimaradiene (1686-66-4)	SPME SD SE	nd a nd a 0.132 ± 0.006 a	nd a nd a 0.574 ± 0.072 b	nd a nd a 0.241 ± 0.212 a	C
dh7	1702	Labda-8(20),12,14-triene (5957-33-5)	SPME SD SE	nd a 0.316 ± 0.007 b nd a	nd a nd a nd a	nd a nd a nd a	C
**Diterpenes (C_20_H_32_) Oxygenated Diterpenes**							
do1	1713	Manoyl oxide (596-84-9)	SPME SD SE	nd a nd a 2.067 ± 0.168 b	nd a nd a 5.627 ± 0.537 c	nd a nd a 1.715 ± 0.328 b	C
Miscellaneous							
m1	2034	Verbenene (4080-46-0)	SPME SD SE	0.061 ± 0.003 b nd a 0.036 ± 0.005 b	nd a nd a nd a	nd a nd a nd a	C
m2	1072	*m*-Cymene (or p-, o-) (535-77-3)	SPME SD SE	0.069 ± 0.004 b 0.085 ± 0.011 b 0.054 ± 0.009 c	nd a nd a nd a	0.186 ± 0.008 c 0.007 ± 0.006 a 0.019 ± 0.006b	C
m3	1082	*p*-Cymene (or m-, o-) (99-87-6)	SPME SD SE	0.261 ± 0.030 a 0.029 ± 0.004 b 0.259 ± 0.229 ab	0.385 ± 0.100 a nd a nd a	4.579 ± 0.192 b 0.198 ± 0.014 c 0.320 ± 0.043 b	B
m4	1775	*Dehydro*-p-cymene	SPME SD SE	0.039 ± 0.007 b nd a 0.075 ± 0.007 b	nd a nd a nd a	0.050 ± 0.006 c nd a nd a	C
m5	1781	*o*-Cymene (or m-, p-) (527-84-4)	SPME SD SE	0.487 ± 0.078 c 0.632 ± 0.066 b 0.629 ± 0.112 b	0.049 ± 0.020 a 0.031 ± 0.008 a nd a	0.201 ± 0.040 b 0.011 ± 0.001 a nd a	C
m6	1795	*exo*-methyl-camphenilol	SPME SD SE	nd a 0.200 ± 0.009 b 0.238 ± 0.023 b	nd a nd a 0.017 ± 0.001 a	nd a nd a nd a	C
m7	1806	Sativens	SPME SD SE	0.097 ± 0.007 b nd a nd a	nd a nd a nd a	nd a nd a nd a	C
m8	1809	α-Abietatriene	SPME SD SE	nd a 0.276 ± 0.027 b 2.947 ± 0.270 b	nd a nd a 3.986 ± 0.752 b	nd a 0.163 ± 0.098 b 0.825 ± 0.468 a	C

^1^ Retention indices (RI) were determined using *n*-paraffins C_7_-C_22_ as external standards on Cyclodex-B column; ^2^ All volatile compounds, positively identified by matching mass spectrum and retention index with those of an authentic standard, are listed by the order of their RI in a chemical class; ^3^ Volatile compounds were calculated with the relative peak ratio of their peak areas to that of internal standard (*n* = 3) ± standard deviation; ^4^ Identification of volatiles was performed requiring the s following criteria: A, mass spectrum and retention index were consistent with those of an authentic standard (positive identification); B, mass spectrum and retention index were consistent with those of literatures [6,20,25]; C^,^ mass spectrum was consistent with that of Wiley 7n spectral database (Agilent Technologies) or by manual interpretation (tentative identification); ^5^ Difference letters mean significant differences (*p*< 0.05) between three different needle samples according to three different cultivar species or extraction methods by Duncan’s multiple range test; ^6^ nd = not detected; ^7^ CAS Registry number.

**Table 4:** Enantiomeric excess (e.e) and configuration of chiral terpenes extracted from three different cultivars based on three different extraction methods.

Volatile Compounds ^1^	Extraction Methods	Enantiomeric Excess(e.e, %) ^2^					
		CP		CO		TO	
		Confgn. ^3^	e.e	Confgn.	e.e	Confgn.	e.e
α-Pinene	SPME	+	86.96	+	90.31	+	71.55
	SD	+	87.89	+	93.91	+	66.91
	SE	+	87.79	+	94.25	+	67.78
Sabinene	SPME		nd ^4^	+/−	100.00	+/−	18.26
	SD		nd	+/−	100.00	+/−	41.51
	SE		nd	+/−	100.00	+/−	38.29
Limonene	SPME	+	49.57	+	91.52	+	24.09
	SD	+	56.70	+	96.74	+	23.29
	SE	+	58.54	+	97.48	+	29.89
β-Phellandrene	SPME	−	100.00	−	100.00	−	100.00
	SD	−	52.92	+	17.25	−	96.68
	SE	−	41.44	−	100.00	−	98.01
4-Terpineol	SPME	−	100.00	−	66.05	+	57.09
	SD	+	12.08	−	100.00	+	4.27
	SE	+	18.95		nd	+	6.65
Bornyl acetate	SPME	−	100.00	−	87.59	−	100.00
	SD	−	100.00	−	75.16	−	55.63
	SE	−	96.59	−	78.93	−	100.00
γ-Muurolene	SPME	+/−	47.84	+/−	100.00	+/−	72.85
	SD		nd	+/−	100.00	+/−	61.79
	SE	+/−	2.51	+/−	13.46	+/−	22.32
Caryophyllene	SPME	−	64.50	−	56.53	−	92.56
	SD	−	100.00	+	100.00	−	91.06
	SE	−	45.95	−	19.85	−	93.47
γ-Curcumene	SPME	+/−	52.10		nd	+/−	51.40
	SD	+/−	100.00		nd	+/−	100.00
	SE		nd		nd		nd

^1^ The enantiomeric isomers separated on Cyclodex-B column; ^2^ The enantiomer excess (e.e, %) = ((predominant enantiomer − minor enantiomer)/(predominant enantiomer + minor enantiomer)) × 100; ^3^ Confgn. = The optical configuration of predominant enantiomer as following criteria: +, (+)-isomers identified by authentic chiral standard or retention index; −, (−)-isomers identified by authentic chiral standard or retention index; +/−, were identified without distinction of chirality; ^4^ nd = not detected.
